# Atrial Fibrillation and Heart Failure

**DOI:** 10.3390/jcm11092510

**Published:** 2022-04-29

**Authors:** Leonard Bergau, Philipp Bengel, Vanessa Sciacca, Thomas Fink, Christian Sohns, Philipp Sommer

**Affiliations:** 1 Clinic for Electrophysiology, Herz- und Diabeteszentrum Nordrhein-Westfalen, Ruhr Universität Bochum, 32545 Bad Oeynhausen, Germany; l.bergau@gmx.de (L.B.); vsciacca@hdz-nrw.de (V.S.); tfink@hdz-nrw.de (T.F.); csohns@hdz-nrw.de (C.S.); 2 Department of Cardiology and Pneumology, Heart Center, University Medical Center, 37075 Goettingen, Germany; philipp.bengel@med.uni-goettingen.de

**Keywords:** atrial fibrillation, heart failure, catheter ablation, remodeling

## Abstract

Atrial fibrillation (AF) is the most common sustained arrhythmia worldwide and has a strong association with heart failure (HF). It often remains unclear if HF is the cause or consequence of AF due to the complexity of the processes that are involved in both the perpetuation of AF and the development of HF. To date, two therapeutic strategies are accepted as the standard of care in AF patients with heart failure. Rhythm control aims to permanently restore sinus rhythm, whereas a rate-control strategy aims to slow ventricular rate without the termination of AF. In the last 5 years a tremendous number of important studies have been published investigating the optimal therapeutic strategy in HF patients. This review highlights the important studies with respect to the involvement of AF in promoting left-ventricular dysfunction and discusses the optimal strategy in HF patients suffering from AF.

## 1. Introduction

Atrial fibrillation (AF) is the most common cardiac arrhythmia worldwide with increasing prevalence and a major impact on societal health and health economy [1].

Therapy for AF follows a multidisciplinary concept, with an emphasis on stroke prevention and symptom relief. To date, two different therapeutic approaches for the treatment of AF are used in common practice—rhythm control and rate control. Whereas rhythm control aims to maintain the sinus rhythm, the intention of rate control is to slow the ventricular rate. In the early 2000s, the AFFIRM trial indicated equality between both concepts regarding their primary endpoints of mortality and stroke [2]. Another study by Roy et al. showed the same in patients with heart failure (HF) [3]. However, in the rhythm control arm of both studies, the use of antiarrhythmic drugs, especially amiodarone, was significantly higher. The potential side effects of chronic amiodarone use are well described [4,5] and might have influenced mortality in these studies. Since the conduction of these studies, the safety and efficacy of rhythm-control strategies have significantly improved, especially by establishing catheter-based isolation of the pulmonary veins (PVI) as a routine strategy in symptomatic AF patients [6]. In contrast to the above-mentioned studies, in recent years evidence has grown to suggest that rhythm control improves outcomes, especially in patients with (HF) [7]. Taking this into account, catheter ablation of AF is currently recommended as a class I indication in most HF patients [6].

This review aims to elucidate the current evidence of AF-related mechanisms leading to heart failure and current therapeutic approaches.

### 1.1. Impact of AF on Atrial Function

**Figure 1:**
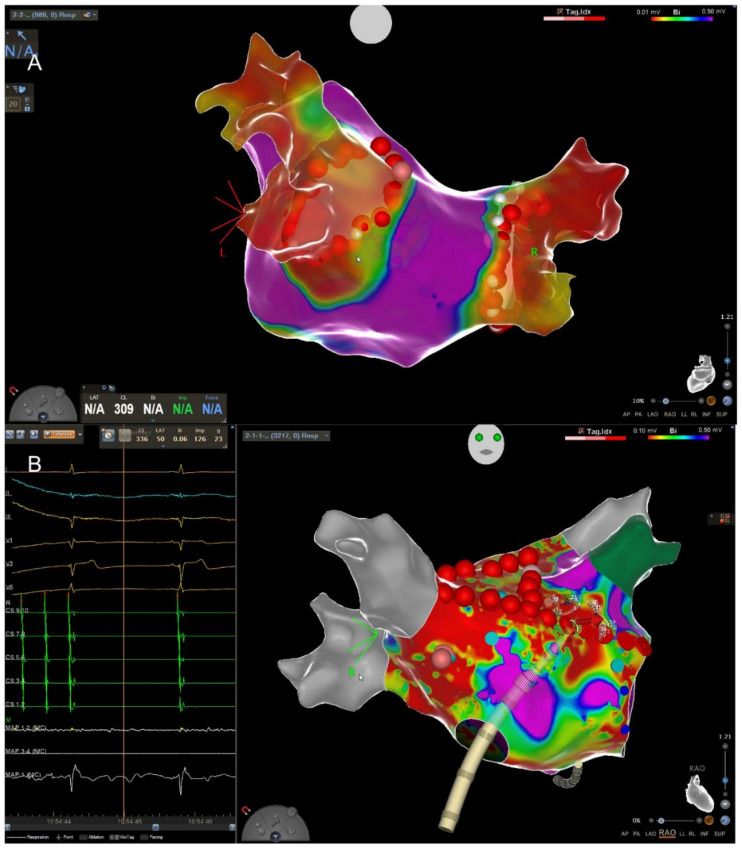
(**A**) High-density 3D map of the left atrium (LA) after pulmonary vein isolation (PVI). View from posterior to anterior. The red dots depict the ablation line encircling the right and left pulmonary veins. The red color indicates that the veins are isolated, and the violet color corresponds to healthy myocardium. (**B**) Termination of atypical flutter through application of a septal line connecting the mitral annulus with the right superior pulmonary vein (RSPV) depicted in grey. The real-time ECG on the left shows the termination into stable sinus rhythm. In contrast to (**A**), the LA shows extensive fibrosis (green = left atrial appendage).

The effect of AF on atrial (LA) function has been well studied. Chronic AF leads to intracellular oxidative stress, with the consequence of calcium overload and onset of the inflammation cascade [8,9]. Altogether, this leads to both the perpetuation of the arrhythmia and the remodeling of the LA, with consequent fibrosis [9]. This chronic process is referred to as “atrial failure” in the literature [10]. It is important to note that atrial fibrosis detected by cardiac MRI significantly influences the success of rhythm-control strategies, especially catheter ablation (Figure 1) [11]. The DECAAF II trial (NCT02529319) investigated the effect of the targeted ablation of fibrotic areas in contrast to PVI only [12]. Its results are eagerly awaited as they might influence further therapeutic strategies.

### 1.2. Impact of AF on Left-Ventricular Function

While the effects of AF on LA myocytes have already been investigated, even on a cellular basis, the AF-associated effects on ventricular myocardium have not yet been sufficiently understood. The MR-guided studies of Kowallick et al. and Prabhu et al. described the negative effects of AF on LV function [13,14]. Both studies showed that patients in AF scheduled for PVI with preserved or impaired LV function showed significantly longer T1 relaxation times as compared with a healthy matched control group. Three to six months after catheter ablation and the restoration of a stable sinus rhythm, the T1 time significantly decreased back to normal values. Since the T1 relaxation time is an early marker for LV fibrosis, it underlines the negative effects of AF on the LV and the potential for reversing the remodeling by establishing a stable sinus rhythm. Soulat-Dufour et al. showed that the restoration of sinus rhythm leads to a reversal of echocardiographic remodeling parameters such as the index volume of both atria, the end-systolic right ventricular volume as well as an improvement in four-chamber function [15]. Besides these observations, it has been shown that the positive effect of betablockers on mortality in HF patients vanishes when the patient is in AF instead of sinus rhythm, even if the AF is rate controlled [16]. This may be due to the remodeling initiated by AF directly counteracting the positive effects of the beta-blocker.

**Figure 2:**
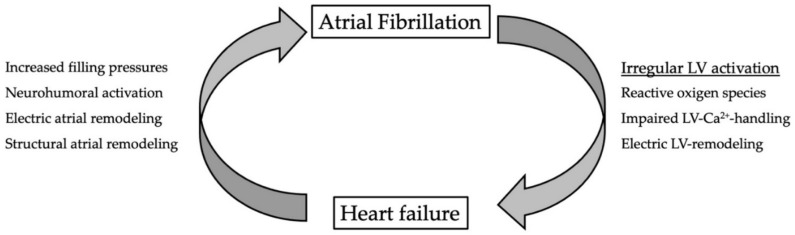
Vicious circle between atrial fibrillation and heart failure. Irregular activation impairs left-ventricular function by intracellular production of reactive oxygen species. This leads to impaired LV-Ca^2+^-handling and electric remodeling. In heart failure, filling pressures are increased and the neurohumoral cascade is activated. Both contribute to electric and structural remodeling of the atria.

A possible explanation for the effects of AF on LV function was presented in a recent experimental study by Pabel et al. where the authors investigated the molecular mechanisms of AF on left-ventricular myocardium from patients with preserved LV function [17]. They compared LV myocardium from patients with sinus rhythm to patients in rate-controlled AF and demonstrated that in AF patients, intracellular LV-Ca^2+^-homeostasis is altered, and action potentials are prolonged (AP) by an augmentation of late sodium current (I_NaL_). They further stimulated cultured cardiomyocytes from non-failing human myocardium as well as human induced pluripotent stem cell cardiomyocytes (iPSC-CMs) over a period of 24 h or seven days. Interestingly, irregular activation led to an elevation of reactive oxygen species (ROS), which activated Ca^2+^/Calmodulin-dependent protein kinase II (CaMKII) by oxidation. This led to impaired Ca^2+^ homeostasis and electrical remodeling of the myocardium. Interestingly, the inhibition of CAMKII or ROS-scavenging with N-acetylcysteine (NAC) ameliorated these effects. It is important to highlight that the AF was simulated with a normal heart rate; therefore, the effect cannot be interpreted as tachycardic cardiomyopathy but as a direct AF-related mechanism. A summary of the mechanisms of AF inducing LV-dysfunction and heart failure promoting the occurrence of AF ist presented in Figure 2 below.

### 1.3. Keep the Rhythm or Slow the Rate? AF Management in Patients with Heart Failure

As discussed above, since the AFFIRM trial, it was for a long time assumed that rate control is equal to rhythm control in HF patients. This has recently been questioned in several studies, leading to a class I recommendation for AF ablation in a majority of HF patients in the recent ESC-AF guidelines [6].

The first study with a major impact on the guidelines was probably the CASTLE-AF trial [18]. This prospective study randomized 363 patients with AF and a left-ventricular ejection fraction (LVEF) ≤ 35% to an interventional arm with rhythm control via catheter ablation or a conservative treatment with antiarrhythmic drugs. The primary endpoint was a composite of death and hospitalization. During the follow-up period, the endpoint occurred in 51 (28.5%) of the patients in the interventional arm. Almost twice as many patients (*n* = 82; 44.6%) reached the endpoint in the conventional arm (*p* < 0.01). Catheter ablation reduced the absolute risk by 16.1%. An improvement in the LVEF (median +8.0%) in the ablation arm compared with an almost unchanged LVEF in the control arm (median +0.2%) also became apparent. The effect was not significant in the subgroup of patients with an LVEF ≤ 25% for which the study was not powered. The CASTLE-HTx-trial aims to elucidate the effect of catheter ablation in this specific patient cohort [19].

The CAMERA-MRI study published in 2017 came to a similar conclusion [20]. In this study, 66 patients with AF and an LVEF ≤ 35% were randomized to catheter ablation or rate control. During the follow-up period of 6 months, there was a significant improvement in LVEF in the ablation arm (mean absolute +18.3%) compared with patients with rate-controlled AF (mean +4.0%). After a period of 4 years, the LVEF was further improved by 16.4 ± 13.3% as compared with a delta LVEF of 8.6 ± 7.6% in the conventional treatment group (*p* < 0.01) [21].

Furthermore, a substudy from the CABANA trial showed a significant reduction in all-cause mortality and a decrease in the AF burden in the subgroup of patients with HF and AF [22]. However, due to several limitations in study design, the results of the CABANA trial can only be interpreted in the light of its limitations in study design and conduction.

The EAST-AFNET 4 trial, published in 2021, investigated the optimal strategy in recently diagnosed AF patients [23]. It was a prospective, randomized study that included a total of 2789 atrial fibrillation patients between 2011 and 2016. The main inclusion criterion was an AF diagnosis not longer than 12 months. Subjects were then allocated 1:1 to rate control (control arm) or rhythm control (therapy arm). The primary endpoint was a composite of cardiovascular death, stroke, hospitalization for heart failure, or acute coronary syndrome. After a median observation time of 5.1 years, the study was stopped after the third interim analysis because of a significant difference between the two groups in favor of the treatment arm. In the control group, 316 patients met the primary endpoint compared to 249 in the therapy group (*p* < 0.05). Rhythm control therapy reduced the risk of reaching the primary endpoint by 22%. In contrast to previous studies, the amiodarone use was relatively low (not more than 20%) in favor of catheter ablation. It can be postulated that with the introduction of catheter ablation, the positive effect of rhythm maintenance becomes clear. The mortality benefit of a rhythm-control-based strategy was further verified in the HF subgroup consisting of 798 patients [24].

In contrast to the CASTLE-AF trial and the CAMERA-MRI trial, which both compared catheter ablation and antiarrhythmic drug therapy, the recently published RAFT trial compared catheter ablation with a rate-control strategy [25]. During the study period between 2011 and 2018 a total of 411 patients were randomized to either of both therapy arms. The primary outcome was a composite of all-cause mortality and all HF events, with a minimum follow up of two years. Although the LVEF (10.1 ± 1.2% vs. 3.8 ± 1.2%, *p* = 0.017), the six-minute walk distance (44.9 ± 9.1 m 27.5 ± 9.7 m, *p* = 0.025), and the quality of life significantly improved through catheter ablation, the study was stopped early due to futility as the primary endpoint did not meet significance. At that time, the primary endpoint occurred in 50 (23.4%) patients in the ablation-based rhythm-control group and 64 (32.5%) patients in the rate-control group (hazard ratio 0.71 95% CI (0.49, 1.03), *p* = 0.066). Despite the early termination of the study, it is still part of the scientific debate, especially due to the borderline significance of the primary endpoint and the lower-than-expected recruitment. One possible explanation might be that 19% of the patients with an LVEF ≤ 45% (N = 116) were CRT carriers and a total of 60 patients in the rate-control group received AV-node ablation and probably CRT implantation (not reported) over the course of treatment. This might be of importance, as Brignole et al. showed in the APAF-CRT trial that AV-node ablation and CRT implantation for rate control is superior to conventional drug-based rate control in patients with HF and permanent AF [26]. They randomized a total of 133 patients to one of the study groups. The trial was also terminated prematurely due to efficacy after a median follow-up of 29 months. At this point, the primary endpoint, all-cause mortality, occurred in seven (11%) patients in the CRT group as compared with 20 (20%) in the drug-guided group (HR 0.26; *p* < 0.01). Based on this result, AV-node ablation and CRT implantation should be the therapy of choice in HF patients when a rate-control strategy is preferred. Table 1 summarizes the findings on outcomes of AF in heart failure patients.

## 2. Conclusions

AF and heart failure constitute two disease entities that are often linked together, as AF facilitates the occurrence of HF and vice versa. The therapy of AF in patients with heart failure is rather complex, but might improve over the time course due to advancement in catheter-based strategies or medical HF treatment. To date, the literature clearly highlights the negative effects of AF on left-ventricular function and the benefits of a rhythm-controlled strategy. Therefore, it seems appropriate that the restoration of sinus rhythm, preferably by catheter ablation, should be the primary goal in HF patients. If sinus rhythm cannot be restored and AF is considered as permanent, the possibility of AV-node ablation and CRT implantation should be discussed with the patient in a timely manner.

## Figures and Tables

**Table 1:** Summary of different clinical trials reporting on outcomes of different treatment strategies in patients with atrial fibrillation and heart failure.

Trial	Inclusion Criteria	Intervention	Rhythm Control Strategy	Primary Endpoint	Follow-Up	Outcome
AFFIRM Wyse et al.	Not HF dependent, 26% with impaired LV function	Anti-arrhythmic drugs vs rate control	Amiodarone, Disopyramide, Flecainide, Moricizine, Procainamide, Propafenone, Quinidine, Sotalol, electrical cardioversion if necessary	All cause mortality	60 month	Neutral
Roy et al.	LV-EF ≤ 35%	Anti-arrhythmic drugs vs. rate control	Amiodaron, Sotalol, Dofetilide & electrical cardioversion if necessary	Cardiovascular death	60 month	Neutral
CASTLE-AF Marrouche et al.	LV-EF ≤ 35%	Catheter ablation vs. Medical therapy (rate or rhythm control)	Catheter ablation (PVI)	Death from any cause or hospitalization for worsening heart failure	60 month	Favors catheter ablation
CAMERA-MRI Prabhu et al.	Idiopathic Cardiomyopathy, LV-EF ≤ 45%	Catheter Ablation vs. Medical Rate Control	Catheter ablation (PVI)	Change in LV-EF	6 month	Favors catheter ablation
CABANA-substudy Packer et al.	Clinically stable heart failure	Catheter ablation vs. Medical therapy (rate or rhythm control)	Catheter ablation (PVI)	Death, Disabling stroke, Serious bleeding, or Cardiac arrest	60 month	Catheter ablation produced clinically important improvements in survival, freedom from AF recurrence, and quality of life relative to drug therapy.
EAST-AFNET 4- substudy Rillig et al.	Heart failure (independent of LV-EF)	Rhythm vs. Rate control	Catheter ablation (PVI), antiarrhythmic drugs, electrical cardioversion if necessary	Cardiovascular death, stroke, or hospitalization for worsening of heart failure or for acute coronary syndrome	72 month	Favors rhythm control
RAFT-Parkash et al.	NYHA II-III, elevated NT-pro-BNP	Catheter Ablation vs. Medical Rate Control	Catheter ablation (PVI)	All cause mortality and all HF events	60 month	Non-significant trend for improved outcomes with ablation-based rhythm control over rate-control
APAF-CRT Brignole et al.	HF-hospitalization in previous year (independent of LV-EF)	Pace and ablate strategy vs. Medical Rate Control	AV-node ablation + CRT-implantation	All cause mortality	48 month	Favors “Pace and ablate”
Chen et al. (Meta-Analysis)	“Heart Failure” not specified	Anti-arrhythmic drugs vs. rate control, Catheter ablation vs rate control, Pooled Analysis	Every Intervention allowed	All-cause mortality, Re-hospitalization, Stroke, and Thromboembolic events	Varying	Favors catheter ablation for rhythm control
